# Physical connectivity simulations reveal dynamic linkages between coral reefs in the southern Red Sea and the Indian Ocean

**DOI:** 10.1038/s41598-019-53126-0

**Published:** 2019-11-12

**Authors:** Yixin Wang, Dionysios E. Raitsos, George Krokos, John A. Gittings, Peng Zhan, Ibrahim Hoteit

**Affiliations:** 1 King Abdullah University of Science and Technology (KAUST), Department of Earth Science and Engineering, Thuwal, 23955-6900 Kingdom of Saudi Arabia; 2 National and Kapodistrian University of Athens, Department of Biology, Athens, Greece; 3 Plymouth Marine Laboratory (PML), Remote Sensing Group, The Hoe, Plymouth, PL1 3DH United Kingdom

**Keywords:** Ecological modelling, Conservation biology, Physical oceanography

## Abstract

The southern Red Sea is genetically distinct from the rest of the basin; yet the reasons responsible for this genetic separation remain unclear. Connectivity is a vital process for the exchange of individuals and genes among geographically separated populations, and is necessary for maintaining biodiversity and resilience in coral reef ecosystems. Here, using long-term, high-resolution, 3-D backward particle tracking simulations, we investigate the physical connectivity of coral reefs in the southern Red Sea with neighbouring regions. Overall, the simulation results reveal that the southern Red Sea coral reefs are more physically connected with regions in the Indian Ocean (e.g., the Gulf of Aden) than with the northern part of the basin. The identified connectivity exhibits a distinct monsoon-related seasonality. Though beyond the country boundaries, relatively remote regions of the Indian Ocean may have a substantial impact on the southern Red Sea coral reef regions, and this should be taken into consideration when establishing conservation strategies for these vulnerable biodiversity hot-spots.

## Introduction

Connectivity refers to the demographic linking between populations across geographically separated regions, through the exchange of individuals, primarily during their pelagic larval stage^1,2^. Connectivity plays a key role in maintaining biodiversity of coral reef ecosystems and enhances their resilience to natural and anthropogenic pressures^2–4^. Connectivity has also become a valuable tool in designing marine protected areas (MPAs), as it provides information and metrics on larval dispersal^2–6^. Various techniques have been developed to investigate connectivity, the most prominent of which being genetic approaches which evaluate connectivity according to the genetic similarity between geographically separated subpopulations^4^. As these linkages are strongly influenced by physical mechanisms such as tides and currents^7,8^, physical connectivity approaches that model larval dispersal have also been widely utilized^9–13^.

The Red Sea is a narrow (maximum width of ~355 km) and elongated (~13°N to ~30°N) marine ecosystem^14^. Coral reef complexes occupy the majority of the Red Sea coastal zone^15^, collectively forming one of the longest coral reef systems on Earth^16^. The mean circulation pattern within the basin is characterized by two vertical meridional overturning cells^17–19^. Coral reef complexes in the Red Sea are mainly influenced by the upper overturning cell, which, on average, consists of a northward flow in the upper layer and a southward flow in the intermediate layer^19^. Despite the narrow width, the Red Sea exhibits a dynamic upper layer circulation associated with strong eddy activity^20,21^, which play an important role in phytoplankton blooms^22,23^ and connectivity^15,24^.

One could expect a genetic homogeneity of marine organisms throughout this narrow and eddy-dominated basin; however, an abrupt genetic break has been identified for various coral reef species at latitudes lower than ~20°N^25–29^, genetically separating the southern Red Sea from the northern part of the basin. Previous studies have revealed homogeneity within the community^30^ and genetic^25–29,31^ structures of the coral reefs in the northern Red Sea (above ~20°N). In contrast, the southern Red Sea exhibits a distinct genetic structure compared with the rest of the basin, as demonstrated by the genetic break observed in reef fishes^25–27^, bivalves^28^ and sponges^29^. For example, a study of the anemonefish *Amphiprion bicinctus* revealed that genetic homogeneity exists in the northern Red Sea, whilst a genetic break occurs at ~19°N and distinguishes the southern end from the rest of the basin^26^. In support of the genetic studies, Raitsos *et al*.^15^ utilized a circulation-driven particle tracking model to show that the southern Red Sea is significantly less connected with the northern basin, and attributed the previously observed genetic break to the local circulation. In addition, as the Red Sea is characterized by pronounced south-to-north environmental gradients (e.g., temperature, salinity and Chl-a), previous studies have also ascribed this genetic break to such heterogeneous environmental conditions^25,26,29^.

The water exchange between the Red Sea and the adjacent Indian Ocean occurs through the Gulf of Aden via the Bab-El-Mandeb strait (hereafter referred to as the ‘strait’) - a narrow strait of ~25 km width^32^. The water influx through the strait is an important nutrient input for the Red Sea^22,23,33–35^ and is characterized by a distinct seasonality^17–19^. During winter, the circulation at the strait consists of two layers: an upper inflow of relatively fresh water and a lower outflow of high salinity water^18,19^. During summer, the circulation pattern changes to a three-layer system, with a fresher inflow at intermediate depths sandwiched between two outflows with higher salinity^17,19^. A previous particle tracking experiment revealed that particles released in the Gulf of Aden near the strait can travel northwards into the southern Red Sea (up to 18°N) within two weeks^36^, indicating the existence of a prominent physical connectivity pathway between these two regions. Moreover, it has been reported that anemonefish specimens from the Gulf of Aden are genetically similar to individuals from the southern Red Sea^25^, suggesting a gene flow that is possibly driven by physical connectivity. In addition, populations in the southern Red Sea were found to be more similar to populations in the Gulf of Aden and Arabian Sea relative to the rest of the basin^37^. Thus, it has been hypothesized that the distinct genetic structure of the southern Red Sea may be explained by the water influx from the Gulf of Aden^15^ (see their supplementary text). However, further research on this hypothesis has been restricted by limited genetic sampling in the Red Sea and the Gulf of Aden, and difficulties in acquiring high-resolution satellite-derived observations of physical circulation in the narrow Bab-El-Mandeb strait. Despite the importance of the southern Red Sea, the factors responsible for this genetic separation still remain unclear.

In this study, we aim to elucidate the important physical connectivity pathways that contribute to the distinct genetic structure of the southern Red Sea. Specifically, we simulate the circulation in the narrow Bab-El-Mandeb strait using a high-resolution regional hydrodynamic model, expanding the research domain toward regions outside the Red Sea (including the Gulf of Aden and the Indian Ocean). Next, we utilize long-term, 3-D backward particle tracking simulations to investigate whether coral reef complexes in the southern Red Sea are more connected with regions outside the Red Sea, rather than with the northern part of the basin.

## Results

**Figure 1:**
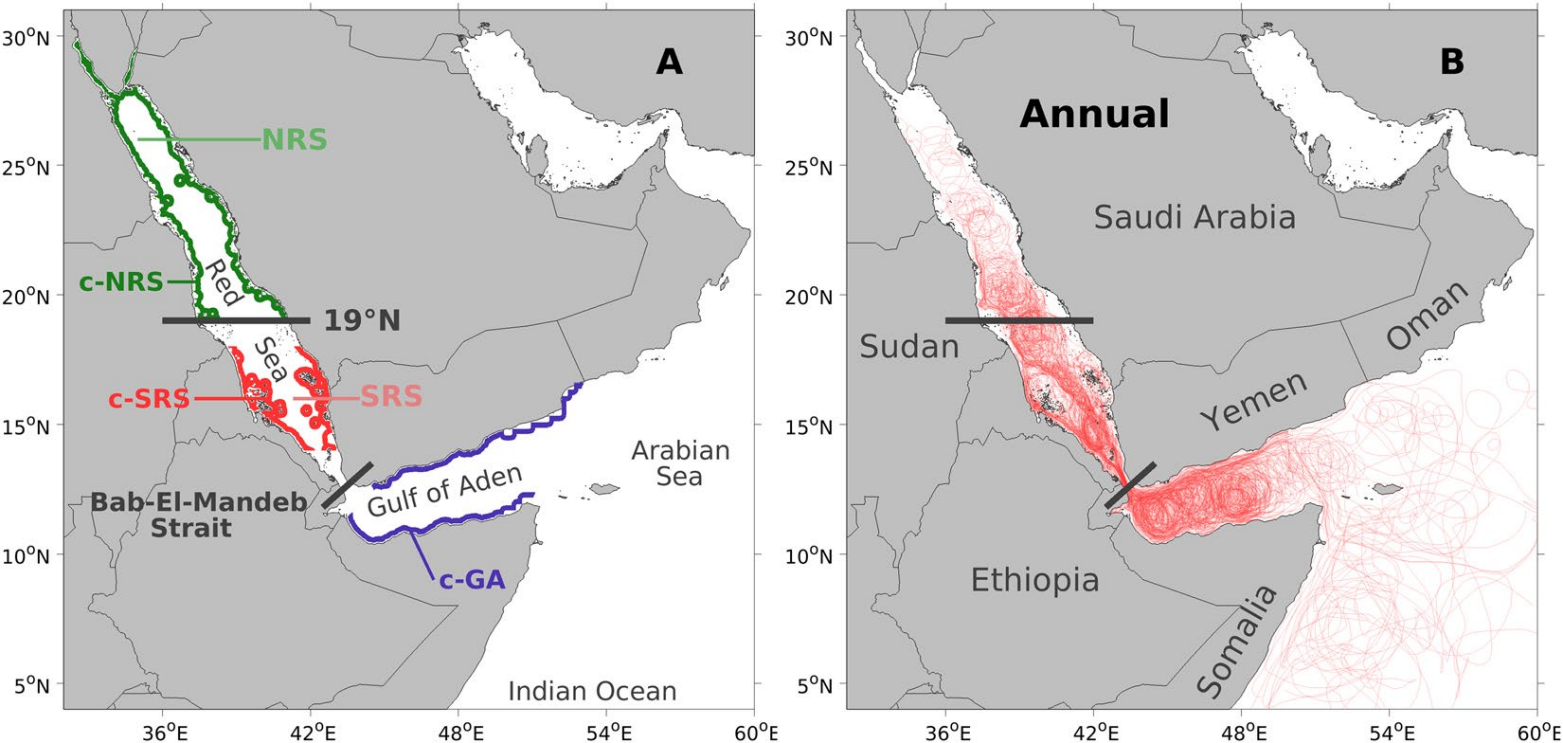
Study regions and annual trajectories. (**A**) Map highlighting the division of the three study regions: the Northern Red Sea (NRS), the Southern Red Sea (SRS), and the regions outside the Red Sea. The NRS refers to the region north of 19°N. The SRS refers to the region between 19°N and the Bab-El-Mandeb strait. The regions outside the Red Sea include the Gulf of Aden, the Arabian Sea and the broader Indian Ocean. For each region, a coastal area is denoted as *c-NRS*, *c-SRS* or *c-GA*, where ‘c’ is short for ‘coastal’ (e.g., the *c-GA* is the acronym for ‘coastal Gulf of Aden’). Note that the *c-SRS* is the coastal area where the backward-tracked particles were released. (**B**) Annual trajectories of 120 randomly selected Lagrangian particles which were released on a monthly basis during the 10-year experiment (each particle representing one releasing month during the 10 years). Particles were released at multiple depths and backward-tracked for 360 days.

To investigate the potential origins of water masses that eventually reach the coral reef assemblages in the southern Red Sea, over 17 million simulated Lagrangian passive particles were released in the coastal southern Red Sea (Fig. 1A, *c-SRS*) and then backward-tracked for 360 days (see methods). The study area was divided into three main regions: the Northern Red Sea (NRS), the Southern Red Sea (SRS), and the regions outside the Red Sea (Fig. 1A).

### Lagrangian particle trajectories

To investigate the impact of the general circulation on connectivity, the annual trajectories of randomly selected Lagrangian particles are presented in Fig. 1B. The particle trajectories show the backward dispersal patterns by providing a spatial representation of the particle movement process, where the dynamics of the trajectories (i.e., their density and abundance) are effective indicators of physical connectivity (Fig. 1B). In the regions outside the Red Sea, the trajectories are evidently dynamic (i.e., dense and abundant), particularly in the Gulf of Aden, and spread throughout the gulf towards the Indian Ocean, where the distribution becomes sparser. In contrast, the trajectories in the NRS exhibit relatively limited dynamics and decrease in intensity as the particles propagate northward. In comparison with the NRS, trajectories in the Gulf of Aden are substantially denser, even though these two regions share a similar distance to the particle releasing site *c-SRS*. Moreover, trajectories in the SRS are generally abundant and exhibit a remarkable advection pattern near the Bab-El-Mandeb strait.

Overall, the annual trajectories demonstrate a significantly more dynamic pattern in the Gulf of Aden compared with the northern Red Sea, highlighting the important influence of the external regions on the coastal southern Red Sea.

### Seasonality of particle trajectories

**Figure 2:**
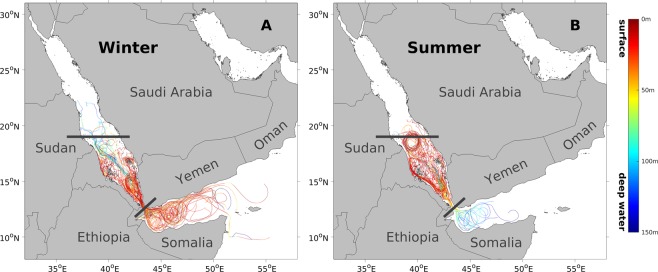
Lagrangian particle trajectories during winter and summer. (**A**) Trajectories of 100 randomly selected particles released in March (representing winter) during the 10-year experiment. Particles were released at multiple depths and backward-tracked for 90 days. Colours indicate the particle depth. (**B**) Same as (**A**) except for showing particles released in September (representing summer).

As the general circulation in the region is characterized by a strong seasonality, we attempted to elucidate the impact of the regional seasonal circulation on the trajectory patterns (Fig. 2). In general, the trajectories during the two seasons reveal substantial differences in terms of their spatial patterns and depth distributions. In the regions outside the Red Sea, the trajectories during winter are shallower, denser and more abundant compared with those during summer (Fig. 2). During winter, trajectories spread throughout the Gulf of Aden with highly dynamic patterns, and are generally constrained within the upper ~50 m layer (Fig. 2A). During summer, trajectories are spatially constrained in the western Gulf of Aden with less dynamic patterns compared to winter, mostly remaining deeper than ~100 m (Fig. 2B). On the other hand, the trajectories in the Red Sea basin (SRS and NRS) exhibit a more complex seasonality (Fig. 2). During winter, notably deep trajectories in the SRS spread northward to the NRS in a dispersive manner, while shallow trajectories exist mainly south of ~18°N (Fig. 2A). During summer, the trajectories inside the Red Sea occur mostly in shallow layers and outline a distinct eddy structure centred at ~18.5°N (Fig. 2B). Regardless of the season, trajectories in the Gulf of Aden are substantially denser compared with those in the NRS (Fig. 2).

Overall, the winter circulation (Fig. 2A) contributes more to the highly dynamic annual trajectories in the regions outside the Red Sea (Fig. 1B), facilitating strong physical connectivity pathways with the Gulf of Aden and ultimately with the Indian Ocean.

### Lagrangian probability density function

**Figure 3:**
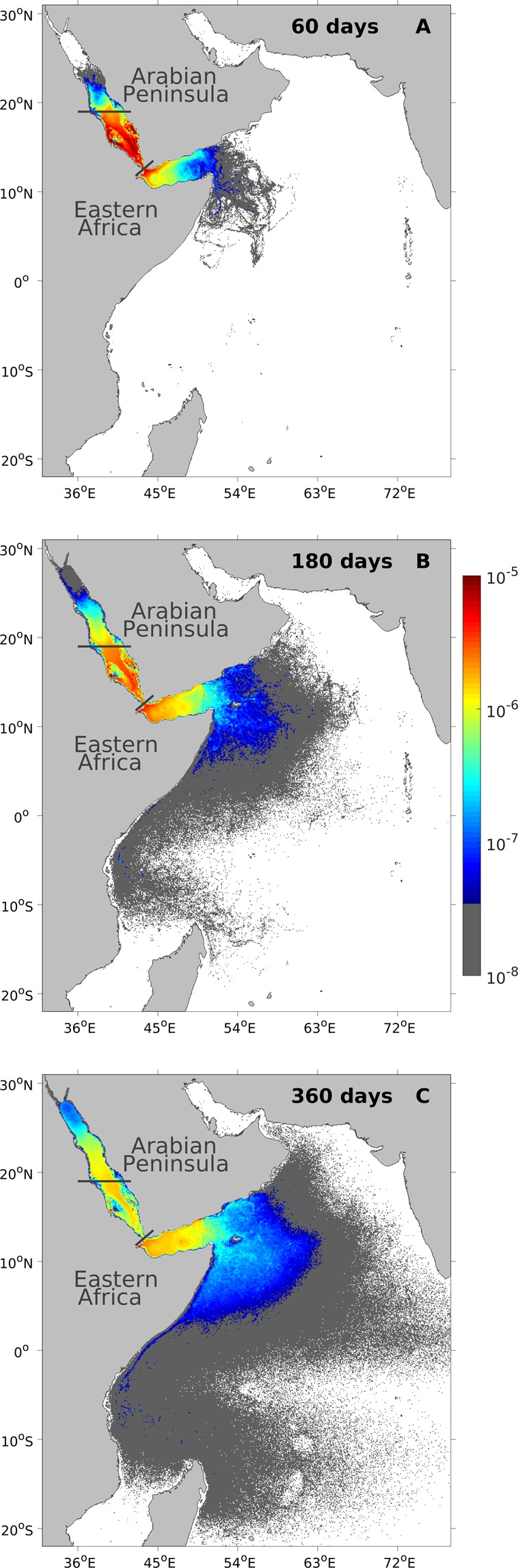
Lagrangian probability density functions (PDFs) of the particles released from the *c-SRS*. The Lagrangian PDFs are backward-tracked for (**A**) 60 days, (**B**) 180 days, and (**C**) 360 days. Colours indicate the probability density of the particle location in km^−2^, where higher values indicate higher levels of connectivity with the releasing site *c-SRS*. Note the logarithmic scale of the colorbar.

To further investigate the backward dispersal patterns, we examined the density distribution of the particles using the Lagrangian probability density function (PDF)^38^. Here, the Lagrangian PDFs at time stages of 60, 180 and 360 days were chosen to investigate the temporal evolution of particle backward dispersal patterns within a calendar year (Fig. 3). Within one simulation year, the dispersal patterns expand to regions as far as the eastern Arabian Sea and the western Indian Ocean.

In the regions outside the Red Sea, backward-tracked particles rapidly occupy the entire Gulf of Aden within 60 days (Fig. 3A). By the end of the simulation year, particles reach regions as far as the Gulf of Oman, the Maldives and Madagascar (Fig. 3C). In contrast, simulated particles that travel to the NRS remain in the central Red Sea for the first 60 days and do not reach the northern part of the basin (Fig. 3A). The particle concentration in the NRS is also generally lower compared with the Gulf of Aden at all three time stages (Fig. 3). In the Arabian Sea and the broader Indian Ocean, the particle concentration is generally low but exhibits a clear tendency to expand more towards the southern Indian Ocean (e.g., Madagascar) rather than towards the north-eastern Arabian Sea (e.g., India and Pakistan). Some regions along the east coast of Africa, such as the east coast of Somalia, exhibit relatively higher particle concentrations, indicating their potential connectivity with the southern Red Sea (Fig. 3).

Overall, the Lagrangian PDFs exhibit a higher particle concentration in the Gulf of Aden compared to the northern Red Sea at the three simulation time stages, once again underlining the important influence of the regions outside the Red Sea. Based on integrated information from all of the simulated particles, the Lagrangian PDFs also highlight the importance of some remote regions like the east coast of Somalia for their potential connectivity with the coastal southern Red Sea.

### Particle fraction time series and source strength

**Figure 4:**
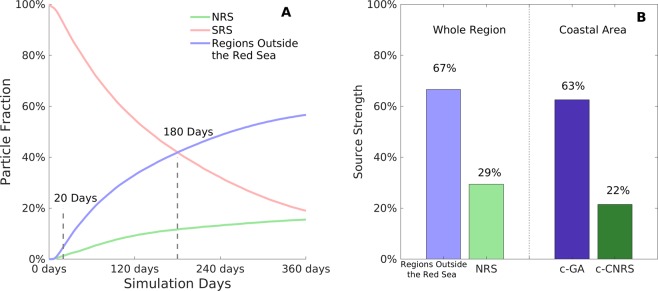
Particle fraction time series and source strength. (**A**) Particle fraction time series of the three study regions computed at all simulation time stages, ranging from 0 to 360 days. (**B**) Source strengths of the regions outside the Red Sea and the northern Red Sea, with ‘whole region’ and ‘coastal area’ results presented separately. For (**A**) and (**B**), higher values indicate higher levels of connectivity with the *c-SRS*.

To further depict the temporal evolution of particle distribution, we next calculated the particle fraction time series for each region (Fig. 4A). The particle fraction refers to the proportion of particles that reside in each study region at a given simulation time. Figure 4A quantitatively describes an increasing importance of the regions outside the Red Sea as the simulation time increases. Evolving monotonically, the fraction of particles residing in the regions outside the Red Sea increases more rapidly in comparison to the NRS, resulting in a significantly higher value by the end of the simulation time (57% for regions outside the Red Sea and 19% for NRS on the 360^th^ day). After 180 days, particles residing in the regions outside the Red Sea are more abundant than those residing in the SRS (i.e., the region where the particles were originally released). We note that even with shorter simulation times, such as 20 days (i.e., a common pelagic larval duration (PLD) of fish species, e.g., 18.1–23.8 days for *Dascyllus aruanus*^39,40^ and 18.9–21.4 days for *Abudefduf sexfasciatus*^40,41^), simulated particles in the regions outside the Red Sea are already more abundant than those in the NRS.

In order to further assess the intensity of the physical connectivity, we also calculated the source strength for each region (Fig. 4B). The source strength describes the ability of a particle originating from each region to reach the coastal southern Red Sea. In order to highlight the most important zone in the regions outside the Red Sea and the NRS, the coastal areas of *c-GA* and *c-NRS* (Fig. 1A) are selected and their source strengths are calculated accordingly (Fig. 4B). Results show an absolute higher possibility for the simulated particles to reach the regions outside the Red Sea compared with the NRS. For instance, within the 360 simulation days, nearly two thirds (63%) of the simulated particles are able to reach the coastal Gulf of Aden, and yet only less than one quarter (22%) are able to reach the coastal northern Red Sea (Fig. 4B).

Overall, throughout the one-year simulation, the regions outside the Red Sea almost permanently accommodate a larger proportion of the total particles compared with the northern Red Sea (Fig. 4A). There is also a higher possibility for a simulated particle to reach areas outside of the Bal-El-Mandeb strait rather than areas north of 19°N (Fig. 4B). These results directly support our hypothesis, by emphasizing a persistent higher level of physical connectivity between the southern Red Sea and the regions outside the Red Sea, in comparison to the northern basin.

## Discussion

In this study, we investigated the circulation-driven physical connectivity of the genetically distinct southern Red Sea, with the aim of identifying the key physical connectivity patterns that can contribute to the observed genetic break. Our backward simulations demonstrate the physical connectivity pathways of the southern Red Sea with its neighbouring regions, highlighting the important connection with the Gulf of Aden and the Indian Ocean. Our results are supported by the observed high level of genetic similarity of anemonefishes between the southern Red Sea (Farasan Islands) and the Gulf of Aden (Djibouti)^25^, and are also in agreement with the population similarity found between the southern Red Sea and the Arabian Sea^37^. Here, a comprehensive understanding of the local circulation characteristics is crucial for interpreting the identified physical connectivity features.

Driven by strong buoyancy forcing and monsoon winds, the annual mean circulation at the Bab-El-Mandeb strait is characterized by a substantial water influx into the Red Sea in the upper layers, and a persistent deep water outflow towards the Gulf of Aden in the bottom layer^17–19^. The water influx regulates the fertility of the southern Red Sea and exhibits a monsoon-related seasonality^22,23,33–35^. During winter, the south-easterly winds prevail over the southern Red Sea^42^ and facilitate the northward surface intrusion in the upper ~90 m above the deep outflow layer^18,19^. This surface intrusion consists of less saline, cooler, relatively nutrient-rich Gulf of Aden Surface Water (GASW), which is important for the winter phytoplankton blooms in the nutrient-depleted southern Red Sea^22,23,33^. During summer, the monsoon winds dominating the southern Red Sea reverse and become north-westerly, facilitating a surface outflow in the upper ~50 m^17,19^. Meanwhile, strong westerly winds over the Gulf of Aden generate upwelling along the northern coastline of the Gulf of Aden, inducing a subsurface intrusion in the intermediate layer (between ~50–100 m) through the strait and into the Red Sea^43,44^. This subsurface intrusion, consisting of fresher, colder and nutrient-rich Gulf of Aden Intermediate Water (GAIW), is sandwiched between the surface and deep outflows of the Red Sea water^17,19^. The GAIW is reported to travel northward up to ~19–22°N^34,45^ (close to the observed genetic break) and plays an essential role in the productivity of the southern Red Sea, through the regulation of summer phytoplankton blooms and the provision of nutrients to the local coral reef ecosystems^34,35^.

Our results reveal that the water influx through the Bab-El-Mandeb strait is a powerful transporter of water masses, connecting the southern Red Sea with the Gulf of Aden (Figs 1–4). The vigorous eddy field in the gulf also facilitates this water mass transport by moving Indian Ocean water closer to the strait area^46,47^ and ultimately into the Red Sea (Fig. 1B). These water masses may act as a carrier of organisms during their pelagic stage, such as larvae. Thus, the water mass transport through the strait could lead to gene flow, and eventually to genetic similarities between the southern Red Sea and the Gulf of Aden/Indian Ocean. Overall, the highly dynamic physical circulation in the strait area (intense water influx) and the gulf (vigorous eddies) leads to a strong physical connectivity between the southern Red Sea and the regions outside the Red Sea. Our results further reveal that this physical connectivity is characterized by a seasonality and intensifies during winter. As indicated by the higher dynamics of the winter trajectories in the Gulf of Aden, the physical connectivity with the regions outside the Red Sea is mostly supported by the surface intrusion of GASW during winter (Fig. 2). In contrast, the subsurface intrusion of GAIW during summer contributes relatively less, despite its high intensity (exhibiting a velocity of up to 0.5 m/s^17^) and important ecological role for the Red Sea (Fig. 2). The observed seasonality of physical connectivity implies that the timing of spawning for different species could impact their actual connectivity pathways due to the change of circulation patterns. For example, larvae that are spawned during winter in the Gulf of Aden might be transported into the southern Red Sea more easily than those that are spawned during summer.

The physical connectivity between the northern Red Sea and the southern basin seems to rely more on the local eddy field (Fig. 1B). Driven by turbulent wind stress (in the southern basin) and isopycnal tilting (in the northern basin), the vigorous mesoscale eddies in the Red Sea exhibit a significantly stronger kinetic energy compared to the local mean general circulation^20,21^. It has been reported that eddies in the Red Sea have a strong ability to transport water masses zonally and rapidly between the east and west coasts^15^. Our study further reveals that in addition to the zonal transport, the meridional transport of water masses from the northern Red Sea to the southern part of the basin is also strongly associated with the local eddies, as indicated by the anti-cyclonic/cyclonic trajectory structure in the Red Sea (Fig. 1B), which is also in agreement with the findings of Zhan *et al*.^48^. However, compared with the intense water influx from the Gulf of Aden, eddies in the Red Sea exhibit a relatively weaker ability to transport water masses (Figs 1B, 3 and 4), which might lead to an insufficient gene flow between the southern and northern parts of the basin, ultimately contributing to the genetic break in the Red Sea.

Within the Red Sea basin, the circulation pattern is also characterized by seasonality. During winter, the deep trajectories in the Red Sea reveal the spreading of the Red Sea Outflow Water (RSOW)^19,49^ (Fig. 2A). The RSOW is formed in the northern Red Sea via convection during winter and flows southward within the intermediate layer, with the outflow rate peaking in winter (February)^19^. As the surface circulation in the central Red Sea during winter is characterized by the northward propagation of the GASW intrusion^19,50^, the water transport to the southern Red Sea is minimal in the upper layer, and is mostly dependent on the spreading of the RSOW in the intermediate layer. During summer, the basin-scale eddy structure centred at ~18.5°N is one of the dipolar eddies spinning up in response to the Tokar Wind Jet at around 19°N (Fig. 2B). This anti-cyclonic eddy is capable of transporting GAIW across the basin with a speed of up to 1 m/s^51^. As indicated by the summer trajectories, this eddy facilitates the zonal water transport between the east and west coasts, but also inhibits the meridional transport between the southern and northern parts of the basin (Fig. 2B). The observed seasonality of physical connectivity within the Red Sea also implies that the timing of spawning for different species could impact their actual connectivity pathways. For instance, the larvae that are spawned during summer in the northern Red Sea are likely to be blocked by the eddy at ~18.5°N and generally do not reach the southern Red Sea.

Our results also reveal potential connectivity pathways between the southern Red Sea and more remote regions in the Indian Ocean (including the Arabian Sea), such as the coasts of Somalia, Socotra and Oman (Fig. 3, Supplementary Figs S1 and S2). The potential occurrence of weaker physical connectivity pathways with the coasts of Kenya, Tanzania and Madagascar is also detected (Fig. 3, Supplementary Figs S1 and S2). The Indian Ocean exhibits a complicated monsoon-driven circulation pattern^52^, which impacts the physical connectivity pathways. For example, the northward Somali Current during summer and the permanent northward East Africa Coastal Current^52,53^ could contribute to the high particle concentration observed along the coasts of southern Somalia and Kenya (Fig. 3C), suggesting the relatively important influence of these regions on the coastal southern Red Sea. The overall monsoon-driven circulation pattern in the Indian Ocean favours a more intense transport of water masses from the western Indian Ocean, in comparison with the northern Arabian Sea (Fig. 3). Thus, from a long-term physical connectivity perspective, the regions along the east coast of Africa may be connected with the coastal southern Red Sea, and a genetic similarity between these regions could be expected.

It has to be acknowledged that, in order to capture the overall circulation-driven connections between the southern Red Sea and different neighbouring regions, our research explored a physical connectivity, rather than simulating species-specific larval behaviours. Generally, connectivity is strongly influenced by physical circulation, as numerous marine larvae disperse nearly passively with local currents, even though larval behaviours like vertical migration and horizontal swimming may also play an important role^1,4,10,15,54,55^ (see “Potential biases introduced by larval behaviours” in methods section). Larval behaviours are difficult to predict and different species exhibit distinct larval behaviours, including PLD and spawning time^1^, which reduces the feasibility of species-specific biological simulations. Here, to explore the Red Sea genetic break that has been observed for various species, we investigated the physical connectivity that was only driven by ocean circulation. Thus, the simulated particles were defined as totally passive and drifted with the local currents so we could inspect the role of physical circulation exclusively. Supporting our approaches, a recent study has reported a remarkable consistency between physical connectivity and genetic population data, demonstrating that physical circulation features formulate gene flow pathways among distant coral reef communities in the Red Sea^15^. The latter study provides us the confidence in the physical circulation approaches’ capacity to investigate coral reef connectivity. However, future genetic studies in the Red Sea, Gulf of Aden and Indian Ocean are still needed to further investigate these model-detected physical connectivity patterns and explore our hypothesis.

Coral reefs constitute some of the world’s most productive ecosystems and are important economic assets through their provision of commercial and artisanal fisheries, recreation and tourism^56^. Effective descriptions of physical connectivity are an important aspect in coral reef conservation^15^. Until now, the reasons behind the observed genetic break in the Red Sea remained unclear. Here, our model-derived physical connectivity provides a possible explanation for the basin-scale genetic heterogeneity. Our simulations reveal that the water masses eventually reaching the coral reef assemblages in the southern Red Sea are more likely to originate from the regions outside the Red Sea, rather than from the northern part of the basin. As the water masses may carry organisms during their pelagic stage, such as larvae, we suggest that the regional circulation might be facilitating the gene flow coming from the regions outside the Red Sea, while obstructing the gene flow coming from the northern basin, which could ultimately explain the observed genetic break. Based on our analysis, we would also expect the southern Red Sea to exhibit a higher level of genetic homogeneity with the Gulf of Aden than with the northern basin. Furthermore, it is possible that genetic similarities occur between the southern Red Sea and more remote regions in the Indian Ocean (e.g., east coast of Somalia). Our research therefore provides a possible explanation for the unique genetic landscape of the southern Red Sea, and brings new insights into the management of the Red Sea and nearby biodiversity hot-spots. As a recent global ecological connectivity study highlighted, areas beyond national jurisdiction (ABNJ) are directly influencing marine ecosystems of coastal countries, and ocean management units should transcend jurisdictional boundaries^55^. Here we show that in addition to the Gulf of Aden, relatively remote regions in the Indian Ocean also have a substantial influence on coral reefs in the southern Red Sea. Thus, effective and sustainable management of these biodiversity hot-spots should be conducted under a cooperative scheme between the countries involved.

## Methods

### Datasets

The backward particle tracking model was implemented based on a nested 3-D velocity field, which consists of a high-resolution (0.01°) MIT general circulation model (MITgcm) simulated dataset and a coarse-resolution (0.25°) Mercator Ocean (Toulouse, FR) GLORYS2V4 reanalysis dataset. The high-resolution MITgcm simulated dataset provided the circulation in the Red Sea and the Gulf of Aden, particularly resolving the water exchange through the strait of Bab-El-Mandeb. The research domain was extended to the Arabian Sea and the north-western Indian Ocean with the coarse-resolution GLORYS2V4 reanalysis dataset, which enabled the model to track particles in a larger spatial scale with an acceptable computing cost.

#### Coarse resolution datasets

The Mercator Ocean (Toulouse, FR) GLORYS2V4 reanalysis product provided an eddy permitting (0.25°) global ocean simulation, acquired from the Copernicus Marine Environment Monitoring Service (CMEMS) (http://marine.copernicus.eu/services-portfolio/access-to-products/?option = com_csw&view = details&product_id = GLOBAL_R EANALYSIS_PHY_001_025). All 75 unevenly spaced vertical layers from the surface (0.5056 m) to the deep sea (5902.0583 m) were included with a horizontal spatial coverage of 20°E-90°E and 30°S-35°N. Daily velocity data from 2002 to 2012 were used to investigate a long-term (>10 yrs) climatological physical connectivity. Since the GLORYS2V4 provided only 3-D horizontal velocity, the vertical velocity *w* was calculated based on the continuity equation under the assumption of an incompressible fluid, with an integration from the bottom (where *w* = 0) to a given depth *z*.

#### High resolution datasets

The high-resolution dataset was simulated by an extensively validated configuration of the MITgcm^57^ for the Red Sea (e.g. Yao *et al*.^17,18^). The MITgcm simulated the hydrodynamics with a horizontal resolution of 0.01° (around 1 km) in a domain between 30°E-50°E and 10°N-30°N, including the entire Red Sea basin, the Gulf of Suez, the Gulf of Aqaba and the Gulf of Aden. The model was implemented with 50 vertical layers, with the vertical resolution varying between 4 m at the surface and 300 m near the bottom. The open boundary conditions with the Arabian Sea and the north-western Indian Ocean were provided on a monthly basis by the parent global GLORYS2V4 reanalysis product to maintain the consistency at the boundary. The potential influence of the monthly basis open boundary conditions was mitigated by trimming the high-resolution domain by 0.1° from the eastern boundary in the Lagrangian particles simulations. The hydrodynamic model was forced by a downscaled regional atmospheric product covering the Red Sea and the adjacent regions^58^ with spatial resolution of ~5 km. The model was integrated from January 2001 to December 2012. The 3-D daily averaged horizontal and vertical velocity were extracted for this study during a 10-year period between 2002 and 2012.

Of special importance for this study, the model accurately reproduced the seasonality, volumes and the 3-D structure of the water exchange at the Bab-El-Mandeb strait, and the propagation and fate of the exchanged water masses inside the basin^17,18,35^. In fact, the model outputs have previously been used in various Red Sea studies and have been shown to successfully describe the circulation dynamics from the large-scale overturning circulation^17,18^ to the mesoscale vigorous eddy activity^20^. In addition, the model results have also been used effectively in the 3-D ecosystem model^23^ and a larval connectivity model^24^ in the Red Sea.

### Numerical particle tracking

#### Backward tracking

To investigate the influence of different neighbouring regions on the southern Red Sea, we defined the connectivity as the one-way transport (instead of the exchange) of larvae or individuals from different marine populations to the coral reef complexes in the southern Red Sea. Thus, backward tracking of particles released in the *c-SRS* was the most efficient approach to identify the origin of water masses and to further estimate the corresponding impacts of different regions on the southern Red Sea (Fig. 1A). The Lagrangian particle tracking model Connectivity Modeling System (CMS) developed by Paris *et al*.^8^ was used to simulate the physical connectivity in this study. CMS coupled a multiple-nested-grid technique with a stochastic Lagrangian framework, which allowed the model to track a large number of particles seamlessly over multiple independent ocean model domains^8^.

#### Experiment design

The Lagrangian particles were released in the coastal southern Red Sea (*c-SRS*) every 1/60 of a degree in a horizontal direction and every 5 meters in a vertical direction at depths between 2 m and 97 m (or the bottom). Released on the 15^th^ of each month during the 10-year simulation period, the particles were tracked backward in time for 360 days, with their locations being calculated every 4 hours and recorded daily. 143,880 particles were released every month, yielding a total number of 17,265,600 for the full simulation period, which was sufficient to resolve the spatial patterns of the Lagrangian PDF. The particles were treated as passive individuals and, to ensure the continuation of the simulation, were re-inserted into the ocean from the latest location once they reached the coastline. The bathymetry data were extracted from the 1/60 degree GEBCO product.

The particle releasing site *c-SRS* is the area defined by a latitudinal range between 14°N and 18°N and an off-coast distance with a width of up to 0.25° (Fig. 1A). To avoid any potential bias introduced by adjacency, the *c-SRS* was at least 1° away from the northern Red Sea and the regions outside the Red Sea. The distance of 0.25° was a reasonable limit for the coral reef complexes in the southern Red Sea. The other coastal areas in Fig. 1A and Fig. S1 were defined according to the same off-coast criterion for consistency with the *c-SRS*. Due to the varying bathymetry of the study area (e.g., extremely steep continental shelf in the Gulf of Aden and mildly decreasing shelf in the Red Sea), a distance criterion was a more reasonable choice than a depth criterion for the selection of coastal areas.

A 360-day simulation experiment was carried out to investigate the physical connectivity in relation to the long-term circulation. To explore the genetic break that results from the long-term re-spawning across generations of coral reef species, our research focused on long-term connectivity patterns instead of a single spawning event. In addition, due to the large spatial scale of our study area, we could not obtain meaningful information on physical connectivity using a simulation period that is too short. On the other hand, an excessively long simulation period would lead to uniformity in the whole study area. Thus, the 360-day simulation time is a compromise, which also takes into account the re-spawning across generations for coral reef species with a short PLD. A 360-day simulation time also covers the PLDs of most reef species (generally up to 1 year with an extreme of 18 months)^13,55,59,60^, enabling the simulation results to provide an overall circulation-driven connectivity reference of a single spawning event for most reef species (Fig. 4A). For instance, for species with a common PLD of 20 days^39–41^, the physical connectivity implies that larvae which are settled in the southern Red Sea coral reefs are more likely to originate from the regions outside the Red Sea, rather than from the northern part of the basin (Fig. 4A). However, since the physical connectivity is only driven by circulation, dispersal patterns might vary with different larval behaviours.

#### Potential biases introduced by larval behaviours

To investigate the circulation-driven physical connectivity, simulated particles were defined as passive in this work. Yet, marine larvae in the ocean exhibit different levels of autonomous behaviours depending on the species^54,61–64^. For example, the larvae of some hard corals exhibit limited swimming abilities during the whole PLD, and thus can be regarded as drifting passively with local currents^62^. In contrast, some reef fish larvae possess remarkable swimming abilities that substantially influence their dispersal, and this should be considered carefully in biophysical modelling^61,65–70^. Larval behaviours involve multiple aspects (e.g., swimming, schooling), of which the vertical migration and horizontal swimming abilities have the most significant impacts on the larval dispersal^61^. Vertical migration indirectly influences dispersal by allowing the larvae to access different circulation regimes with varying current velocities^61,63,69^. Meanwhile, horizontal swimming ability is also important as the larvae of some species might swim faster than the ambient current^61,63,69^. The simulation of larval behaviours is receiving increasing attention in biophysical dispersal modelling studies^65–68,70^.

We acknowledge that to explore the observed genetic break for various species in the Red Sea, we investigated the circulation-driven physical connectivity where the simulation of larval behaviours were omitted and particles were defined as passive. Previous studies using biophysical models have revealed that the simulation of larval behaviours may alter the model results^65,66,70^. On the other hand, Raitsos *et al*.^15^ have also reported a remarkable consistency between the long-term circulation-driven physical connectivity and the genetic structure of a reef fish species (*Amphiprion bicinctus*) in the Red Sea, revealing the important role of physical circulation in forming the genetic structure of a species with potentially active larval behaviours (no larval behaviour data are yet available for the *Amphiprion bicinctus* in the Red Sea, though the similar species *Amphiprion melanopus* has been reported to exhibit considerable larval swimming abilities^69,71^).

### Quantifying connectivity

#### Lagrangian PDF

The Lagrangian PDF describes the probability density distribution of the simulated particle location, and has been widely used to predict the dispersal pattern^38^. In short, the values (in km^−2^) of Lagrangian PDFs are representative of the particle concentrations. Based on Mitarai *et.al*.^38^, we computed the discrete Lagrangian PDF without Gaussian filtering using the equation:

1 $$Lagrangian\,PDF(\xi ,t)=\frac{{n}_{\xi }(t)}{{S}_{\xi }\ast N},$$

where *ξ* is the sample space related to the discretion of Lagrangian PDF (here, a sample space of *∼*86 *km* ^2^ is applied), *S*_*ξ*_ is the area of sample space *ξ*, *N* is the total number of Lagrangian particles, and *n*_*ξ*_(*t*) is the number of particles residing in the sample space *ξ* at the simulation time *t* (*t* ≤ total simulation time *τ*, here *τ* = 360 days).

#### Particle fraction and source strength

The particle fraction of each study region was calculated as the fraction of particles that reside in the region at the simulation time *t* over the total number of Lagrangian particles. Essentially, particle fraction is the spatial integration of the Lagrangian PDFs in each region. In the context of backward particle tracking simulations, the source strength of each study region was calculated as the fraction of the particles that ever reach the region within the total simulation time *τ* over the total number of Lagrangian particles. Note that a particle that has reached the region *r*_*i*_ at the simulation time *t*_1_ may also reach another region *r*_*j*_ at the simulation time *t*_2_, thus the source strengths of all regions may have a sum higher than 1. Also note that the southern Red Sea, including the *c-SRS*, is the particle releasing region and thus its source strength is equal to 1.

## Supplementary information

Supplementary Information

## Data Availability

The Mercator Ocean (Toulouse, FR) GLORYS2V4 reanalysis dataset used in this study is freely available at http://marine.copernicus.eu. The MITgcm simulated dataset is available from the corresponding author on reasonable request.
