# Supplementary material for: Physical connectivity simulations reveal dynamic linkages between coral reefs in the southern Red Sea and the Indian Ocean
Source: Sci Rep. 2019 Nov 12;9:16598. doi: 10.1038/s41598-019-53126-0 (PMC6851178; doi:10.1038/s41598-019-53126-0)
Supplement: Supplementary file 1 — Supplementary Information [file 41598_2019_53126_MOESM1_ESM.pdf]

Physical connectivity simulations reveal dynamic linkages between coral reefs in the southern Red Sea and the Indian Ocean

Yixin Wang[1], Dionysios E. Raitsos[2, 3], George Krokos[1], John A. Gittings[1], Peng Zhan[1], Ibrahim Hoteit[1*]

[1] King Abdullah University of Science and Technology (KAUST), Department of Earth Science and Engineering, Thuwal, 23955-6900, Kingdom of Saudi Arabia

[2] National and Kapodistrian University of Athens, Department of Biology, Athens, Greece

[3] Plymouth Marine Laboratory (PML), Remote Sensing Group, The Hoe, Plymouth, PL1 3DH, United Kingdom

*Correspondence should be directed to Ibrahim Hoteit (ibrahim.hoteit@kaust.edu.sa).

Supplementary Information

This part contains 2 supplementary figures.

Supplementary Figure S1:

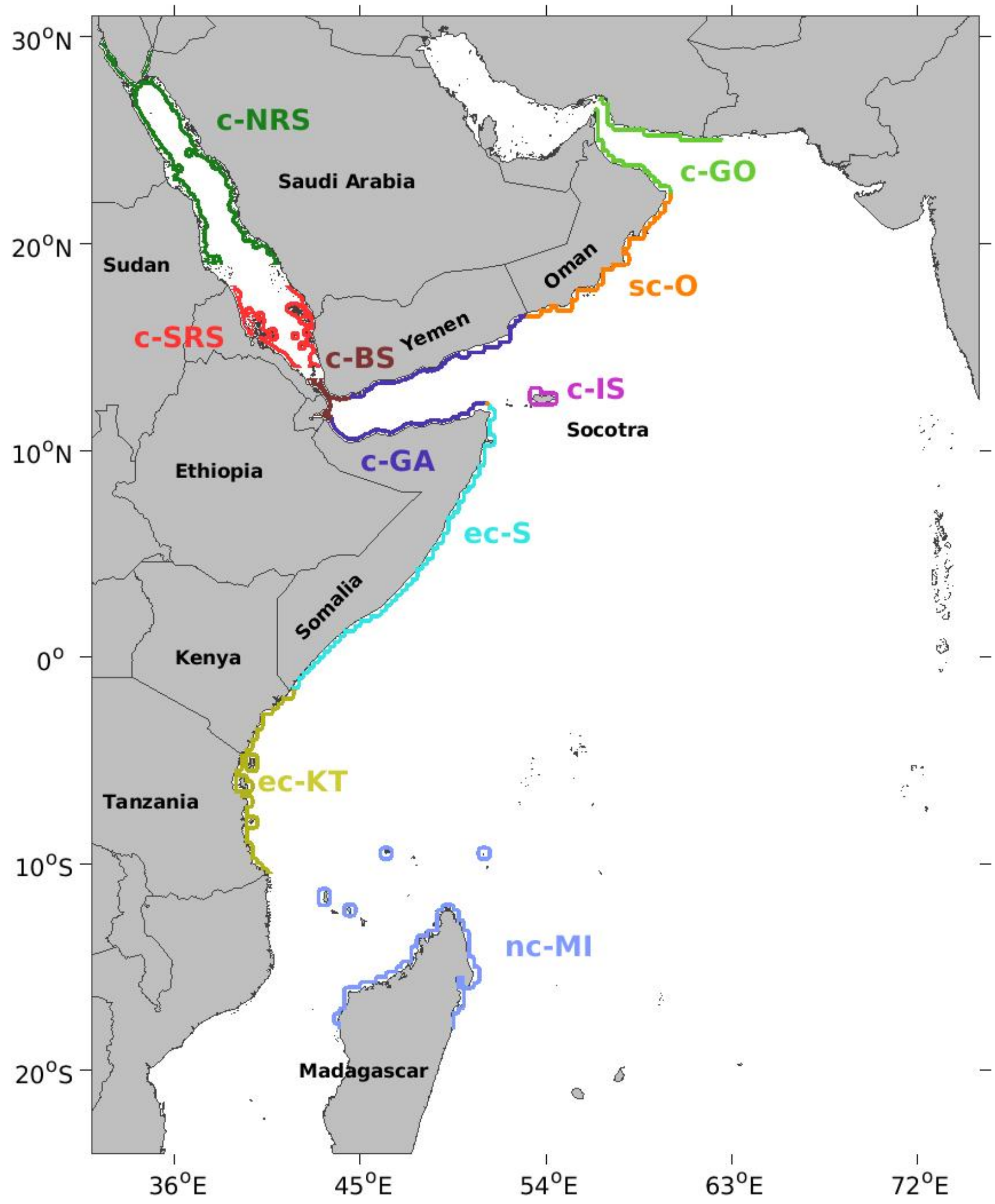

Figure S1. Selection of the ten coastal areas where the backward-tracked particles were released (*c-SRS*) or could possibly reach. In addition to the three coastal areas shown in Fig. 1A, seven more coastal areas were selected to analyze their physical connectivity with the coastal southern Red Sea. Acronyms: *c-NRS* for coastal northern Red Sea, *c-SRS* for coastal southern Red Sea, *c-BS* for coastal Bab-El-Mandeb strait, *c-GA* for coastal Gulf of Aden, *c-IS* for coast of Island Socotra, *ec-S* for east coast of Somalia, *ec-KT* for east coasts of Kenya and Tanzania, *nc-MI* for north coasts of Madagascar and nearby Islands, *sc-O* for south coast of Oman, and *c-GO* for coastal Gulf of Oman.

Supplementary Figure S2:

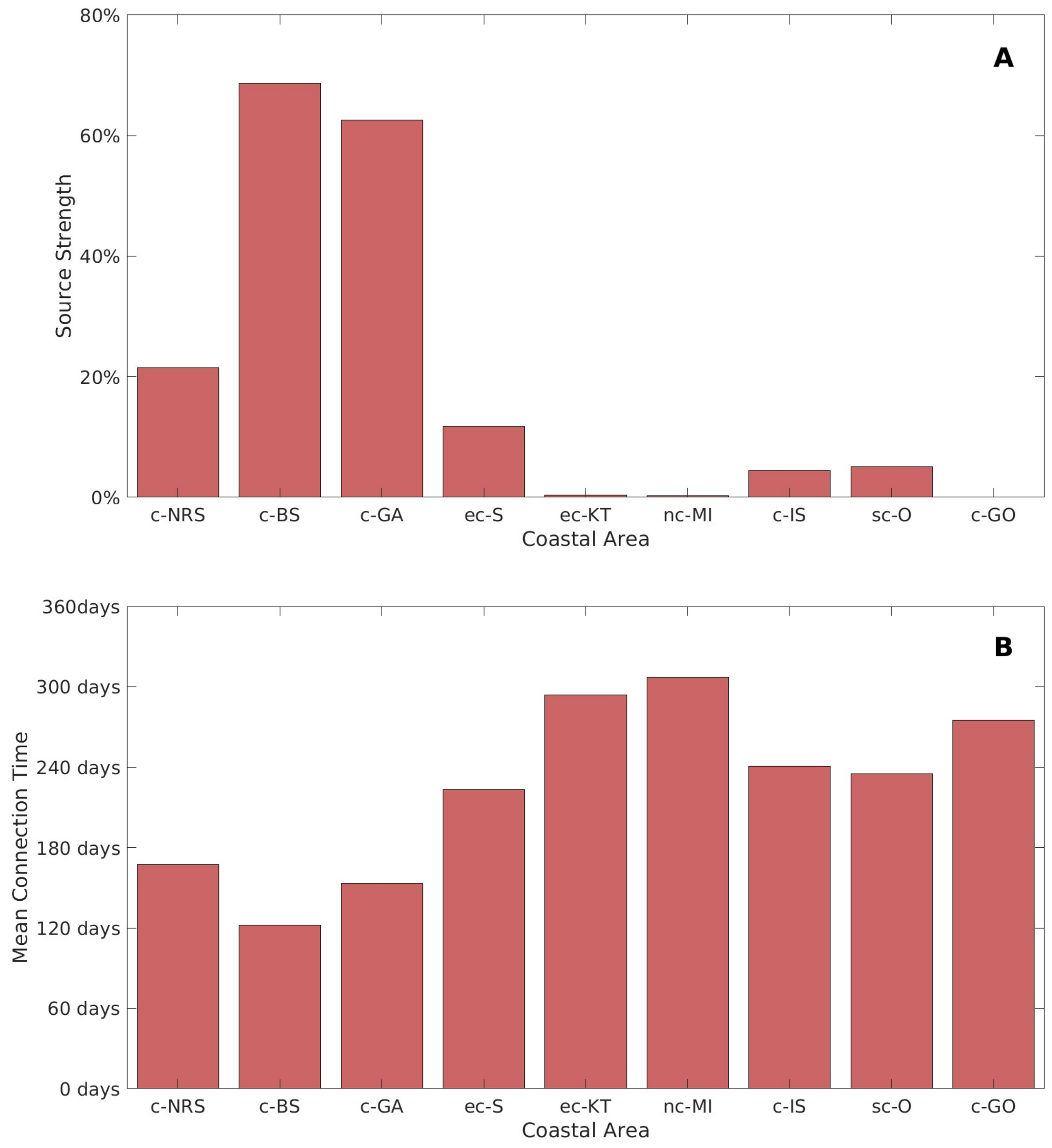

Figure S2. Connectivity metrics of source strength and Mean Connection Time (MCT) for nine coastal areas. (**A**) Source strength and (**B**) MCT. The MCT was calculated as the average time for the particle released in the coastal southern Red Sea to reach a certain coastal area, where a higher value indicates a lower level of connectivity. Acronyms: *c-NRS* for coastal northern Red Sea, *c-SRS* for coastal southern Red Sea, *c-BS* for coastal Bab-El-Mandeb strait, *c-GA* for coastal Gulf of Aden, *c-IS* for coast of Island Socotra, *ec-S* for east coast of Somalia, *ec-KT* for east coasts of Kenya and Tanzania, *nc-MI* for north coasts of Madagascar and nearby Islands, *sc-O* for south coast of Oman, and *c-GO* for coastal Gulf of Oman.
